# An evaluation of the quality of online perinatal depression information

**DOI:** 10.1186/s12884-021-04320-4

**Published:** 2022-03-15

**Authors:** Madison P. Hardman, Kristin A. Reynolds, Sarah K. Petty, Teaghan A. M. Pryor, Shayna K. Pierce, Matthew T. Bernstein, Patricia Furer

**Affiliations:** 1 Department of Psychology, University of Manitoba, 190 Dysart Rd, Winnipeg, MB R3T 2N2 Canada; 2 Department of Clinical Health Psychology, University of Manitoba, Winnipeg, MB Canada

**Keywords:** Perinatal depression, Internet, Information quality, Informed decision making

## Abstract

**Background:**

During the perinatal period (including pregnancy and up to 12 months after childbirth), expectant and new mothers are at an elevated risk of developing depression. Inadequate knowledge about perinatal depression and treatment options may contribute to the low help-seeking rates exhibited by perinatal people. The Internet can be an accessible source of information about perinatal depression; however, the quality of this information remains to be evaluated. The purpose of this study was to assess the quality of perinatal depression information websites.

**Methods:**

After review, 37 websites were included in our sample. To assess overall website quality, we rated websites based on their reading level (Simple Measure of Gobbledegook; SMOG), information quality (DISCERN), usability (Patient Education Materials Assessment Tool; PEMAT), and visual design (Visual Aesthetics of Website Inventory; VisAWI).

**Results:**

Websites often exceeded the National Institute of Health's recommended reading level of grades 6–8, with scores ranging from 6.8 to 13.5. Website information quality ratings ranged from 1.8 to 4.3 out of 5, with websites often containing insufficient information about treatment choices. Website usability ratings were negatively impacted by the lack of information summaries, visual aids, and tangible tools. Visual design ratings ranged from 3.2 to 6.6 out of 7, with a need for more creative design elements to enhance user engagement.

**Conclusions:**

This study outlines the characteristics of high-quality perinatal depression information websites. Our findings illustrate that perinatal depression websites are not meeting the needs of users in terms of reading level, information quality, usability, and visual design. Our results may be helpful in guiding healthcare providers to reliable, evidence-based online resources for their perinatal patients.

## Background

The perinatal period, spanning from pregnancy to 12 months postpartum, can be a joyous time for expectant and new mothers; however, physical, emotional, and lifestyle changes in this period can heighten their risk for developing depression. The Internet can be an accessible source of mental health information for pregnant and postpartum people experiencing depression. Online health information is most often accessed by women and is a popular medium of information consumption for people in the pregnancy and postpartum periods [1–4]. Further to this, previous research suggests that perinatal people who endorse symptoms of depression are more likely to access online mental health resources than those with little to no symptoms [5]. Prior website evaluation research within the area of perinatal mental health has shown that websites vary in quality, are difficult to read, and often contain incomplete information [6, 7]. The quality of online information about perinatal depression has yet to evaluated, which was the focus of this study.

Depression in the perinatal period is common, with prevalence estimates ranging from 11 to 20% across studies [8, 9]. Factors contributing to the development of perinatal depression may include insufficient sleep [10], individual and family mental health history [11], perceived lack of social support [10, 11], and a history of adverse childhood experiences, including abuse [12]. Of concern, perinatal depression can have detrimental effects on both maternal and infant health if left undiagnosed and untreated [11, 13–17]. Despite the prevalence and adverse impacts of perinatal depression, help-seeking rates are low due to a multitude of barriers [18]. For example, pregnant and postpartum people struggling with depression may face criticism or feel ashamed if their perinatal experiences deviate from societal expectations, which often depict this period as full of bliss and satisfaction [19]. Consequently, people in the perinatal period may avoid seeking help for their mental health [19]. Another help-seeking barrier is low mental health literacy, an individual’s knowledge about mental disorders and treatment options, which can hinder mothers’ ability to recognize depression and make informed treatment choices [19, 20].

There are important implications for the use of the Internet to increase understanding and recognition of symptoms of perinatal depression and related treatment options. The Internet can help users gather health-related information outside of medical appointments, assisting the public to make informed treatment decisions [21, 22]. Within a perinatal context, pregnant and postpartum people may use the Internet to independently locate health information, to get a second opinion, and to exert greater control over the decisions affecting their health and that of their child [4]. Nevertheless, it is unclear how well Internet users can discern the quality of online health information [23]. Further, women in the perinatal period often deem online health information to be reliable, but do not always verify their findings with their healthcare provider [24].

Several factors contribute to higher quality health information websites, including readability, information quality, usability, and visual design. The National Institute of Health recommends that health information have a reading level of grades 6–8 [25]; however, online health materials often exceed this recommendation [26, 27]. This is consistent with previous mental health website evaluations, with findings that websites differed greatly in quality [6, 28]. At present, the quality of perinatal depression websites has yet to be assessed, with past evaluations focused solely on depression in the postpartum period [6]. A previous evaluation of perinatal anxiety websites found that most websites had high reading levels, low to moderate information quality, and low actionability ratings [7]. The aim of this study was to evaluate the quality of perinatal depression information websites, with a specific focus on readability, information quality, usability, and visual design.

## Methods

### Procedure

**Fig. 1:**
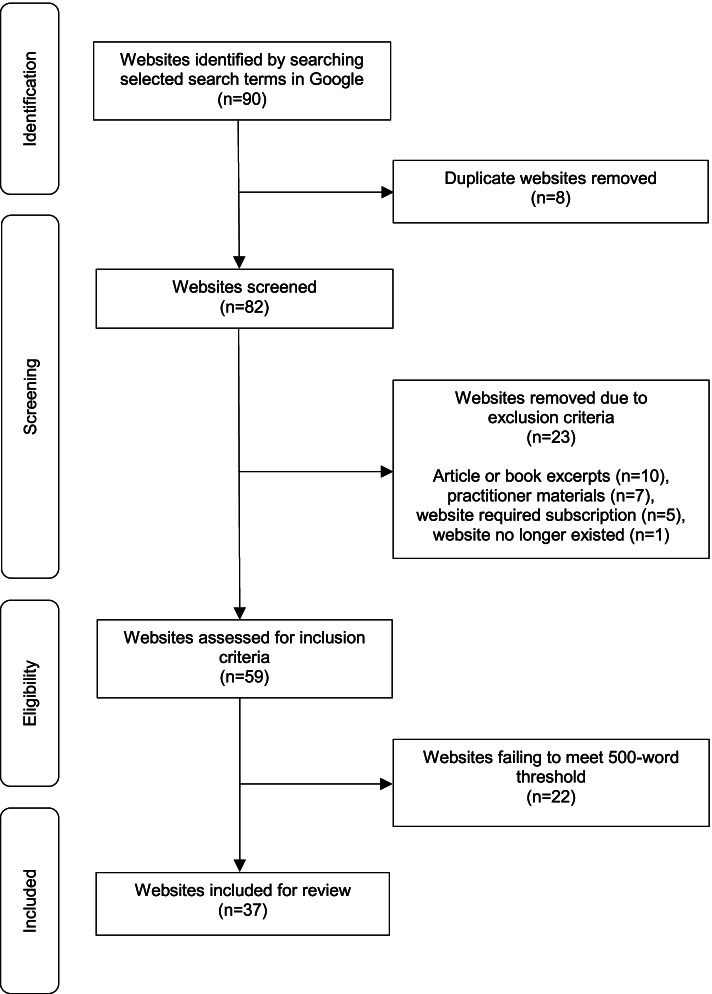
Website selection process

We identified websites for this evaluation by searching three sets of terms in the search engine Google. We selected Google as over 70% of online searches are conducted using this search engine [29]. Prior to each search, we activated Google’s incognito mode and cleared the browsing data, including search history, cookies, and cached files, to reduce the impact of past searches on our search results. To investigate which search terms would return the broadest set of relevant results, we searched several terms related to perinatal depression in Google in February 2020. Based on these results, we selected several lay terms for depression in pregnancy and postpartum, as well as the medical term perinatal depression. Consequently, we captured a wide range of websites that pregnant and postpartum people with depression may visit. Our final search terms included: perinatal depression, depression AND pregnant, and sad after pregnancy. To meet inclusion criteria, websites needed to include at least 500 words on perinatal depression (depression occurring in pregnancy and/or within 12 months postpartum), be written in English, and be retrieved from the first three pages of the search, as most people do not look beyond this [30]. Websites were excluded if they were duplicates, blogs, forums, commercial websites, practitioner materials, book or article excerpts, or were inaccessible to the public (e.g., required a login). As detailed in Fig. 1, 37 websites met inclusion criteria and were evaluated in the current study.

After selection, non-rater SK Pierce took screenshots of each website in March 2020. SK Pierce then de-identified screenshots and randomized the order in which each of our raters (MPH and SK Petty) assessed each website. This procedure was adopted to blind raters to websites in our sample, with the aim of achieving more objective website ratings. Raters reviewed deidentified screenshots and completed independent DISCERN, PEMAT (Patient Education Materials Assessment Tool), and VisAWI (Visual Aesthetics of Website Inventory) ratings for each website. Raters only consulted one another when rating the first three websites to ensure that they had a shared understanding of all scale items.

### Measures

#### Reading level

We calculated website readability using the Simple Measure of Gobbledegook (SMOG) evaluation [31]. This measure indicates the years of education a reader needs to understand the material, known as reading level. When interpreting scores, higher SMOG scores correspond to higher reading levels. We calculated SMOG scores by inputting 30 sentences from each website into an online readability tool [32]. This measure has been used to evaluate the reading level of online materials for a range of mental health conditions, including but not limited to anxiety, depression, bipolar disorder, and schizophrenia [33, 34].

#### Information quality

We assessed website information quality using the DISCERN, a standardized 16-item instrument rated on a scale of 1 (no, does not meet website quality criteria) to 5 (yes, meets website quality criteria) [35]. Raters answer questions about elements related to health information quality, such as whether the information is relevant. The DISCERN has high levels of reliability and validity and has been used to assess online information quality for several mental and physical health topics, including but not limited to perinatal anxiety, posttraumatic stress disorder, and chronic kidney disease [7, 35–37].

#### Usability

We evaluated website usability using the Patient Education Materials Assessment Tool (PEMAT). This measure assesses the understandability and actionability of health information and has high levels of internal consistency, reliability, and construct validity [38]. Understandability is a 19-item evaluation, while actionability is a 7-item assessment. Our two raters independently rated each item with either 0 (Disagree) or 1 (Agree). Materials are considered understandable or actionable if they reach a threshold of 70% or more on each measure [38]. The PEMAT has been used to assess the understandability and actionability of websites focused on various mental and physical health topics, including but not limited to perinatal anxiety, cervical cancer screening, and hypertension [7, 39, 40].

#### Visual design

We assessed website visual design using the Visual Aesthetics of Website Inventory (VisAWI), a standardized 18-item instrument designed for online materials [41]. Raters are prompted to answer questions related to various design elements using a 7-point Likert scale (1 = strongly disagree to 7 = strongly agree). We calculated a general factor of visual aesthetics for each website by averaging ratings for all items [41]. The VisAWI has been used to evaluate visual aesthetics of websites on topics such as anxiety and nutrition [33, 42].

### Analysis

Following previous research, we averaged all 16 items of the DISCERN to produce an overall information quality rating for each website [7, 43]. We also calculated mean ratings and 95% confidence intervals for each DISCERN and VisAWI item. To determine whether mean DISCERN scores were significantly associated with SMOG scores, PEMAT ratings, and mean VisAWI ratings, we calculated Pearson correlation coefficients. As well, we computed correlations with these variables and search engine order from all three searches to evaluate how quality differed across searches. We also calculated interrater agreement for DISCERN, PEMAT, and VisAWI ratings using an interclass correlation coefficient. To make comparisons across measures, we assigned websites a rating (good, adequate, or poor) for each domain evaluated. We then calculated aggregate ratings for all websites and used these to assign websites an overall rank. Table 1 outlines the criteria for good, adequate, and poor classifications for website domain and aggregate ratings. Websites remained blind to evaluators until we completed data analysis.

**Table 1:** Criteria used to rank websites

Measure	Element assessed	Maximum possible score	Classification criteria		
			Good	Adequate	Poor
DISCERN	Information quality	5	> 4	3–4	< 3
SMOG	Readability	N/A	6–8	9–10	> 10
PEMAT	Understandability	100%	> 70%	50–70%	< 50%
	Actionability	100%	> 70%	50–70%	< 50%
VisAWI	Visual design	7	> 6	4–6	< 4
–	Aggregate rating	15	≥ 12	9–11	≤ 8

*SMOG* Simple Measure of Gobbledygook, *PEMAT* Patient Education Materials Assessment Tool, *VisAWI* Visual Aesthetics of Website Inventory

## Results

### Highest rated websites

Table 2 shows website rankings, domain ratings, and aggregate ratings. Only five websites in our sample achieved aggregate ratings falling within the good range, as per the criteria outlined in Table 1. The websites with the highest aggregate ratings were the American Family Physician and the National Health Service, which both had aggregate ratings of 13. Both websites were rated highly in terms of information quality, with the latter having the highest DISCERN rating across websites in our sample. Both websites also met the 70% threshold for understandability and actionability. The American Family Physician had a reading level of 7, thus falling within the recommended reading levels of 6–8; however, the National Health Service exceeded the recommended range, with a reading level of 10. These two websites also varied in terms of their visual design ratings. The American Family Physician had one of the lowest VisAWI ratings in our sample at 3.5, while the National Health Service had a VisAWI rating of 5.4, which falls within the adequate range.

**Table 2:** Perinatal depression website characteristics and dimension comparison – February, 2020

Website	Search engine order	Readability	Information quality	Usability		Visual design	Aggregate rating
				Understandability	Actionability		
Search: perinatal depression							
**Beyond Blue (AUS)**	**5**	**9.0 (A)**	**3.2 (A)**	**89 (G)**	**57 (A)**	**6.6 (G)**	**12 (G)**
Healthy Children (USA)^a^	8	9.7 (A)	3.2 (A)	79 (G)	57 (A)	5.4 (A)	11 (A)
Office on Women’s Health (USA)	14	7.7 (G)	3.9 (A)	68 (A)	43 (P)	6.1 (G)	11 (A)
Mayo Clinic (USA)^a^	4	9.7 (A)	3.4 (A)	68 (A)	57 (A)	5.8 (A)	10 (A)
Healthy Children (USA)^a^	6	8.3 (G)	2.3 (P)	79 (G)	29 (P)	5.5 (A)	10 (A)
Reach Out (AUS)	11	8.2 (G)	1.9 (P)	74 (G)	43 (P)	5.2 (A)	10 (A)
Centre for Addiction and Mental Health (CAN)	12	11.0 (P)	4.2 (G)	58 (A)	29 (P)	4.6 (A)	9 (A)
Canadian Mental Health Association (CAN)	17	10.0 (A)	3.1 (A)	58 (A)	29 (P)	5.7 (A)	9 (A)
American Psychiatric Association (USA)	20	9.8 (A)	3.4 (A)	58 (A)	43 (P)	5.1 (A)	9 (A)
Wikipedia (USA)	23	12.1 (P)	4.0 (G)	63 (A)	29 (P)	4.1 (A)	9 (A)
Healthline (USA)	1	9.7 (A)	2.7 (P)	53 (A)	29 (P)	5.3 (A)	8 (P)
Zero to Three (USA)	13	10.6 (A)	2.2 (P)	63 (A)	29 (P)	5.8 (A)	8 (P)
Very Well Mind (USA)	16	12.9 (P)	3.6 (A)	68 (A)	29 (P)	5.1 (A)	8 (P)
University of North Carolina (USA)	29	11.9 (P)	1.8 (P)	47 (P)	14 (P)	4.3 (A)	6 (P)
Search: depression AND pregnant							
**National Health Service (UK)**^**a**^	**23**	**10.0 (A)**	**4.3 (G)**	**79 (G)**	**71 (G)**	**5.4 (A)**	**13 (G)**
**March of Dimes (USA)**	**9**	**8.0 (G)**	**3.6 (A)**	**74 (G)**	**57 (A)**	**5.8 (A)**	**12 (G)**
Government of Canada (CAN)	4	9.5 (A)	3.4 (A)	89 (G)	57 (A)	5.3 (A)	11 (A)
Parents (USA)^a^	18	9.6 (A)	4.2 (G)	79 (G)	43 (P)	4.9 (A)	11 (A)
Caring for Kids (CAN)	2	6.8 (G)	2.5 (P)	74 (G)	29 (P)	5.6 (A)	10 (A)
Tommy’s (UK)	10	8.8 (G)	3.4 (A)	58 (A)	43 (P)	5.7 (A)	10 (A)
Web MD (USA)^a^	11	8.3 (G)	3.8 (A)	58 (A)	43 (P)	4.0 (A)	10 (A)
Medical News Today (UK)^a^	21	10.5 (A)	3.7 (A)	74 (G)	14 (P)	4.9 (A)	10 (A)
American Pregnancy Association (USA)	5	10.6 (A)	3.3 (A)	68 (A)	43 (P)	4.1 (A)	9 (A)
PSYCOM (USA)	6	10.6 (A)	3.1 (A)	58 (A)	43 (P)	4.3 (A)	9 (A)
Mother and Baby (UK)	28	10.6 (A)	2.3 (P)	74 (G)	43 (P)	4.7 (A)	9 (A)
Mayo Clinic (USA)^a^	3	10.9 (A)	3.1 (A)	53 (A)	43 (P)	3.6 (P)	8 (P)
Everyday Health (USA)	24	13.5 (P)	3.3 (A)	53 (A)	14 (P)	4.8 (A)	8 (P)
Harvard University (USA)	15	11.4 (P)	3.4 (A)	42 (P)	14 (P)	3.7 (P)	6 (P)
Search: sad after pregnancy							
**American Family Physician (USA)**	**11**	**7.0 (G)**	**4.2 (G)**	**74 (G)**	**86 (G)**	**3.5 (P)**	**13 (G)**
**National Health Service (UK)**^**a**^	**4**	**8.5 (G)**	**3.7 (A)**	**79 (G)**	**57 (A)**	**5.3 (A)**	**12 (G)**
Help Guide (US)	9	10.5 (A)	3.7 (A)	68 (A)	57 (A)	5.9 (A)	10 (A)
Medical News Today (UK)^a^	26	9.9 (A)	4.1 (G)	68 (A)	29 (P)	5.4 (A)	10 (A)
National Childbirth Trust (UK)	29	7.5 (G)	2.7 (P)	68 (A)	43 (P)	6.0 (G)	10 (A)
Parents (USA)^a^	15	11.0 (P)	3.8 (A)	84 (G)	29 (P)	4.5 (A)	9 (A)
Web MD (USA)^a^	6	9.9 (A)	2.4 (P)	53 (A)	29 (P)	4.1 (A)	8 (P)
KidsHealth (USA)	2	9.0 (A)	2.2 (P)	47 (P)	43 (P)	4.1 (A)	7 (P)
American College of Obstetrics and Gynecology (USA)	17	9.0 (A)	2.8 (P)	68 (A)	29 (P)	3.2 (P)	7 (P)

Each website was rated on each dimension as Good (G), Adequate (A), and Poor (P), defined differently for each measure. Readability was measured with the Simple Measure of Gobbledygook (SMOG); information quality was measured with the DISCERN; usability was measured with the Patient Education Materials Assessment Tool (PEMAT); visual design was measured with the Visual Aesthetics of Website Inventory (VisAWI). The top scoring websites are indicated in bold type.

^a^Different webpages by the same author were assessed.

Furthermore, three other websites had aggregate ratings falling within the good range. These websites were Beyond Blue, March of Dimes, and a second webpage from the National Health Service, which all had aggregate ratings of 12. With regards to reading level, March of Dimes and the National Health Service fell within the recommended range of grades 6–8, with scores of 8 and 8.5 respectively. Beyond Blue was slightly above the recommended range, with a reading level of 9. These websites all met the 70% threshold for understandability but did not meet criteria for actionability. Further, the DISCERN ratings for these websites only fell into the adequate range based on our criteria outlined in Table 1. Beyond Blue had the highest VisAWI rating across websites in our sample and fell within the good range with a rating of 6.6. On the other hand, March of Dimes and the National Health Service only had VisAWI ratings falling within the adequate range (5.8 and 5.3 respectively).

### Reading level

Websites varied greatly in their reading levels, with ratings ranging from 6.8 to 13.5. Only 10 websites in our sample had reading levels that fell within the recommended range. To determine whether reading level increased with search engine order, we calculated two-tailed Pearson correlation coefficients for all searches. A significant positive association was found between search engine order (search: perinatal depression) and reading level, r(14) = .56, *p* = .04. This indicates that as website order increased in this search, so did reading level. These variables were not significantly associated in other searches.

### Information quality

We calculated mean ratings for each DISCERN item to assess website performance across items (Table 3). Website information quality ratings ranged from 1.8 to 4.3 out of 5 (M = 3.2, SD = .7), with mean item ratings varying between 2.6 to 3.9 out of 5. Interrater agreement for the DISCERN was excellent, r(37) = .84, *p* = .01. Mean overall website information quality (item 16) was 3.1, indicating that websites only partially meet DISCERN criteria. Several of the lowest rated items included items 4 (sources of information used; M = 2.8, SD = 1.5) and 5 (when sources were produced; M = 2.7, SD =1.1). The highest rated items were 3 (relevance of content; M = 3.9, SD = .8) and 14 (more than one treatment option provided; M = 3.9, SD = 1.0). With the exception of item 14, all items related to treatment (items 9–13) were rated low to moderate. These items include how treatment works (item 9, M = 2.7, SD = 1.4), the benefits and risks of treatment (item 10, M = 2.6, SD = 1.1; item 11, M = 3.0, SD = 1.4), what happens if no treatment is used (item 12, M = 3.0, SD = 1.4), and how treatments affect quality of life (item 13, M = 3.1, SD = 1.1). We calculated two-tailed Pearson correlation coefficients to determine whether search engine order and mean DISCERN ratings were associated across searches. There were no significant relationships between these variables.

**Table 3:** Mean scores of DISCERN items across all websites

DISCERN item	Mean score	95% confidence interval
1. Are the aims clear?	3.4	[3.1, 3.7]
2. Does it achieve its aims?	3.8	[3.6, 4.0]
3. Is it relevant?	3.9	[3.6, 4.1]
4. Is it clear what sources of information were used to compile the publication (other than the author or producer)?	2.8	[2.3, 3.3]
5. Is it clear when the information used or reported in the publication was produced?	2.7	[2.3, 3.1]
6. Is it balanced and unbiased?	3.4	[3.1, 3.7]
7. Does it provide details of additional sources support and information?	3.3	[2.8, 3.8]
8. Does it refer to areas of uncertainty?	3.2	[2.6, 3.6]
9. Does it describe how each treatment works?	2.7	[2.2, 3.2]
10. Does it describe the benefits of each treatment?	2.6	[2.2, 2.9]
11. Does it describe the risks of each treatment?	3.0	[2.5, 3.4]
12. Does it describe what would happen if no treatment was used?	3.0	[2.6, 3.5]
13. Does it describe how the treatment choices affect the overall quality of life?	3.1	[2.7, 3.4]
14. Is it clear that there may be more than one possible treatment choice?	3.9	[3.6, 4.2]
15. Does it provide support for shared decision making?	3.6	[3.2, 4.0]
16. Based on the answers to all of the above questions, rate the overall quality of the publication as a source of information about treatment choices.	3.1	[2.8, 3.4]

Each DISCERN item is rated on a five-point scale with the anchors 1 (no, does not meet website quality criteria) and 5 (yes, meets website quality criteria). Number on left-hand side denotes item number in scale.

### Usability

Understandability ratings ranged from 42 to 89% (M = 66.7, SD = 11.8), with moderate interrater agreement, r(37) = .61, *p* = .01. Only 14 websites in our sample met the understandability threshold, with most lacking information summaries and visual aids. To determine whether websites with high information quality also had high understandability, we calculated a two-tailed Pearson correlation coefficient. The relationship between these variables was not significant, r(37) = .26, *p* = .13. Moreover, actionability ratings varied greatly (14–86%, M = 40.0, SD = 16.2), with high interrater agreement, r(37) = .76, *p* = .01. Overall, only two websites were actionable, with websites often missing tangible tools. To determine whether information quality and actionability were associated, we computed a two-tailed Pearson correlation coefficient. There was a significant relationship between these variables, r(37) = .34, *p* = .04, indicating that websites with higher information quality also had greater actionability. To assess whether search engine order and actionability were associated across searches, we calculated Pearson correlation coefficients. There was a significant negative relationship between these variables (search: perinatal depression), r(14) = −.55, *p* = .04, indicating that websites with higher actionability ratings appeared earlier in this search. These variables were not significantly associated in the other two searches.

### Visual design

We calculated mean VisAWI item ratings to assess website performance across items (Table 4). Website visual design ratings ranged from 3.2 to 6.6 out of 7 (M = 5.0, SD = .8) with mean VisAWI item ratings ranging from 4.1 to 5.7 out of 7. The highest rated items were related to website layout and use of colour, including items 4 (site appears patchy; M = 5.7, SD = 1.3), 12 (colours do not match; M = 5.6, SD = 1.3), and 13 (choice of colours is botched; M = 5.6; SD = 1.3). Websites received lower ratings on items related to design creativity, including items 6 (design is uninteresting; M = 4.1, SD = 1.6), 7 (layout is inventive, M = 4.4, SD = 1.4), and 8 (design appears uninspired, M = 4.2, SD = 1.2). To determine whether there was a relationship between search engine order and mean VisAWI ratings across searches, we calculated two-tailed Pearson correlation coefficients. There was a significant negative relationship between these variables (search: perinatal depression), r(14) = −.64, *p* = .01, indicating that websites earlier in this search had superior visual designs. These variables were not significantly associated in the other searches.

**Table 4:** Mean scores of VisAWI items across all websites

VisAWI item	Mean score	95% confidence interval
1. The layout appears too dense. (r)	4.7	[4.2, 5.2]
2. The layout is easy to grasp.	5.2	[4.8, 5.6]
3. The layout appears well structured.	5.1	[4.7, 5.5]
4. The site appears patchy. (r)	5.7	[5.2, 6.1]
5. Everything goes together on this site.	5.1	[4.7, 5.4]
6. The design is uninteresting. (r)	4.1	[3.6, 4.6]
7. The layout is inventive.	4.4	[4.0, 4.9]
8. The design appears uninspired. (r)	4.2	[3.8, 4.6]
9. The layout appears dynamic.	5.0	[4.5, 5.4]
10. The layout is pleasantly varied.	4.9	[4.5, 5.3]
11. The colour composition is attractive.	4.9	[4.5, 5.2]
12. The colours do not match. (r)	5.6	[5.1, 6.0]
13. The choice of colours is botched. (r)	5.6	[5.2, 6.1]
14. The colours are appealing.	4.8	[4.5, 5.2]
15. The layout appears professionally designed.	4.8	[4.4, 5.2]
16. The layout is not up-to-date. (r)	5.5	[5.1, 5.9]
17. The site is designed with care.	4.9	[4.5, 5.2]
18. The design of the site lacks a concept. (r)	4.9	[4.6, 5.3]

Each VisAWI item is rated on a 7-point scale with the anchors 1 (strongly disagree) and 7 (strongly agree). Number on left-hand side denotes item number in scale. (r) denotes reverse scored item.

Initially, VisAWI interrater agreement was low due to the broad range of possible responses and the subjectivity of this measure. To improve interrater agreement, raters reassessed by consensus any of their ratings that were two or more points apart for items 1, 6, 7, 8, 12, 13, 14, and 18. We limited reassessment to these items to preserve as many of our independent ratings as possible; however, interrater agreement remained low. In response, raters reassessed by consensus any total VisAWI ratings differing by seven or more, which represented the upper third of the data. This resulted in excellent interrater agreement, r(37) = .97, *p* = .01.

## Discussion

The purpose of this study was to evaluate the quality of perinatal depression information websites, with the literature currently limited to evaluations of perinatal anxiety and postpartum depression websites [6, 7]. Websites in our sample were predominantly of low to moderate quality, with only five websites achieving good aggregate ratings. With regards to readability, only 10 of 37 websites fell within the recommended reading level (grades 6–8).

The websites with the highest overall ratings were the American Family Physician and the National Health Service, with the latter also having the highest information quality rating across websites in our sample. Although the National Health Service had a high reading level of 10, the American Family Physician fell within the suggested range with a reading level of 7. Both websites met criteria for understandability and actionability (70%). Their visual design ratings varied, with the American Family Physician receiving one of the lowest visual design ratings across websites in our sample, while the National Health Service had a visual design rating falling within the adequate range. Other highly rated websites included Beyond Blue, March of Dimes, and a second webpage from the National Health Service. March of Dimes and the National Health Service fell within the recommended readability range, with reading levels of 8 and 8.5 respectively; however, Beyond Blue was slightly above the recommended range with a reading level of 9. Although all three of these websites met criteria for understandability, they did not meet criteria for actionability. Further, their information quality ratings only fell within the adequate range. Beyond Blue had the highest visual design rating across websites in our sample, while March of Dimes and the National Health Service had visual design ratings falling within the adequate range.

It is essential that websites present trustworthy content to ensure that pregnant and postpartum people with depression can make informed treatment choices. Information quality scored lowest in areas related to the sourcing of information and information about treatment options. This aligns with findings of previous mental health website evaluations, suggesting that website authors must incorporate evidence-based sources and convey details of these sources to users [7, 28, 43]. Furthermore, most websites lacked detailed descriptions of treatment benefits and focused primarily on risks; however, information was generally limited to pharmacological interventions, which is consistent with a review of adult depression websites [28]. Websites successfully conveyed relevant information, such as depression symptoms, and provided comprehensive lists of treatment options.

Only two websites in our sample met criteria for both understandability and actionability, which is consistent with a previous evaluation of perinatal anxiety websites [7]. To improve understandability, websites can include summaries of key information and visual aids. The lack of visual materials is problematic, as videos may be an effective means of destigmatizing mental illness [44, 45]. As well, women may prefer greater visual aids when learning about postpartum depression [46]. Websites in our sample also had poor actionability features, including a lack of tangible tools, such as symptom checklists. There was a significant negative correlation between search engine order (search: perinatal depression) and actionability ratings, indicating that websites earlier in these results were more actionable. As well, mean information quality and actionability ratings were positively correlated.

Overall, website visual design ratings varied widely, with most websites in our sample falling within the adequate range. Websites possessed strong structural elements, such as well-designed layouts, in addition to engaging colour choices; however, they often lacked creative design features, such as inspiring design elements. It must be noted that only one of the five top-rated websites in our sample had a visual design rating falling within the good range. Given that perceived aesthetics may influence users’ first impressions of a website and perceptions of trustworthiness, user engagement with online mental health materials may be increased through improved visual design [47, 48]. Within our sample, a significant negative correlation was found between search engine order (search: perinatal depression) and mean visual design ratings, suggesting that websites with superior designs appeared earlier in this search.

### Limitations

This study is additive and complementary to the growing number of mental health website evaluations; however, it is not without limitations. Despite our broad range of search terms, these terms are not reflective of all of the terms that may be used by pregnant and postpartum people experiencing depression, or those close to them who are looking for information and support. Further to this, we recognize that our search terms may not have captured all of the specialized perinatal mental health websites available online. Future research using different search strategies and evaluating additional perinatal mental health websites would be a valuable addition to the extant research in this area. Our search results may also have been impacted by region and may not include all available websites. As well, only websites that were written in English were assessed, which limits the generalizability of our results. Our search data was also limited to the date on which our searches were completed. Other search engines may have produced differing results, however, we followed the precedent of previous website evaluations and only used Google, the most widely used search engine [7, 28, 29, 33, 43].

Additionally, there are several limitations to the methods that we used to rate websites in our sample. Although blinding websites to raters reduced subjective bias, there were drawbacks to this method. Specifically, it was not effective when rating websites such as Wikipedia that have highly recognizable appearances. Further, raters did not rate all VisAWI items independently due to initially low interrater agreement, attributed to the subjectivity of the VisAWI and its broad rating scale. It is important to contextualize our challenges with interrater reliability for the VisAWI within the extant body of research. The literature reveals a range in coefficients across studies measuring interrater agreement for shorter versions of the VisAWI (0.11–0.88) [49, 50]. This highlights the need for consideration on the use and potential refinement of this method to ensure that ratings are consistent across reviewers.

## Conclusion

This study adds to the growing body of literature on mental health website evaluations, and more specifically, evaluations focused on perinatal mental health websites. Overall, websites in our sample varied greatly in quality. Websites often exceeded the recommended reading level, suggesting that website creators must produce more easily understandable content. Furthermore, there was a paucity of treatment-related information, which would hinder users’ ability to make informed treatment choices. Poor understandability and actionability ratings suggest that website usability must be improved, namely by adding information summaries, visual aids, and tangible tools to help users seek support. At present, perinatal depression websites are not meeting the needs of the public in terms of reading level, information quality, usability, and visual design. Our findings may guide healthcare providers, people who are pregnant or postpartum and experiencing depressive symptoms, and their supporters, to high quality online resources focused on perinatal depression. Several high-quality resources that can be referred to perinatal people experiencing depression include the American Family Physician, Beyond Blue, March of Dimes, and the National Health Service.

We recommend that future perinatal mental health website evaluations integrate a variety of medical terms into their searches (e.g., postpartum depression). We expect this would return a different sample of websites, which, in conjunction with the findings presented in this study, may extend researchers’ understanding of the quality of websites focused on mental health in the perinatal period. Future research could also include the use of several different search engines, which may result in a larger sample of websites that could be assessed. Further to this, non-English websites or websites from different regions about perinatal depression could be assessed to determine how the quality of perinatal mental health websites differs across languages as well as geographically.

## Data Availability

The datasets used and/or analysed during the current study are available from the corresponding author on reasonable request.

## Abbreviations

SMOG: Simple Measure of Gobbledegook
PEMAT: Patient Education Materials Assessment Tool
VisAWI: Visual Aesthetics of Website Inventory
